# Selection of reference genes from two leafhopper species challenged by phytoplasma infection, for gene expression studies by RT-qPCR

**DOI:** 10.1186/1756-0500-6-409

**Published:** 2013-10-11

**Authors:** Luciana Galetto, Domenico Bosco, Cristina Marzachì

**Affiliations:** 1 Istituto di Virologia Vegetale, CNR, Strada delle Cacce 73, 10135 Torino, Italy; 2 Dipartimento di Scienze Agrarie, Forestali e Alimentari, Università degli Studi di Torino, Via Leonardo Da Vinci 44, 10095 Grugliasco, TO, Italy

**Keywords:** “*Candidatus* Phytoplasma asteris”, *Euscelidius variegatus*, *Macrosteles quadripunctulatus*, Insect vectors, Housekeeping genes, BestKeeper, geNorm, Normfinder

## Abstract

**Background:**

Phytoplasmas are phloem-limited phytopathogenic wall-less bacteria and represent a major threat to agriculture worldwide. They are transmitted in a persistent, propagative manner by phloem-sucking Hemipteran insects. For gene expression studies based on mRNA quantification by RT-qPCR, stability of housekeeping genes is crucial. The aim of this study was the identification of reference genes to study the effect of phytoplasma infection on gene expression of two leafhopper vector species. The identified reference genes will be useful tools to investigate differential gene expression of leafhopper vectors upon phytoplasma infection.

**Results:**

The expression profiles of *ribosomal 18S*, *actin*, *ATP synthase β*, *glyceraldehyde-3-phosphate dehydrogenase* (*GAPDH*) and *tropomyosin* were determined in two leafhopper vector species (Hemiptera: Cicadellidae), both healthy and infected by “*Candidatus* Phytoplasma asteris” (chrysanthemum yellows phytoplasma strain, CYP). Insects were analyzed at three different times post acquisition, and expression stabilities of the selected genes were evaluated with BestKeeper, geNorm and Normfinder algorithms. In *Euscelidius variegatus*, all genes under all treatments were stable and could serve as reference genes. In *Macrosteles quadripunctulatus*, BestKeeper and Normfinder analysis indicated *ATP synthase* β, *tropomyosin* and *GAPDH* as the most stable, whereas geNorm identified reliable genes only for early stages of infection.

**Conclusions:**

In this study a validation of five candidate reference genes was performed with three algorithms, and housekeeping genes were identified for over time transcript profiling of two leafhopper vector species infected by CYP. This work set up an experimental system to study the molecular basis of phytoplasma multiplication in the insect body, in order to elucidate mechanisms of vector specificity. Most of the sequences provided in this study are new for leafhoppers, which are vectors of economically important plant pathogens. Phylogenetic indications were also drawn from sequence analysis of these genes.

## Background

Phytoplasmas, wall-less plant pathogenic bacteria belonging to the Class Mollicutes, are classified as “*Candidatus* Phytoplasma spp.”, infect a wide variety of plants and cause significant economic losses worldwide [1]. In the infected plants, phytoplasmas are restricted to phloem elements and cause growth disorders, leaf and floral alterations, eventually leading to plant death. The pathogenicity mechanisms are still unclear, but some nucleus-targeted virulence factors secreted by phytoplasma cells alter plant metabolism, playing a crucial role in symptom development and insect vector interaction [2]. Phytoplasmas have a very small, A-T rich genome ranging from 530 to 1350 kb [3]. The genome is generally organized as a circular chromosome [4-6], but it is linear in “*Ca.* P. mali” [7]. In phytoplasma genomes, several multi-copy genes are organized in clusters of potential mobile units (PMUs), flanked by a transposase gene and inverted repeats, probably involved in host adaptation [8].

Phytoplasmas are transmitted in a persistent and propagative manner by phloem-feeding insects in the Order Hemiptera. A latent period in the vector is required by phytoplasmas to colonize the insect body, including salivary glands, and be transmitted [9]. Insect vector specificity plays a key role in the epidemiology of these pathogens, and phytoplasmas are usually transmitted by a narrow range of vector species [10], while their plant host range is usually broader [11]. For this reason, identification of the molecular determinants of vector specificity is crucial to understand the epidemiology of important phytoplasma diseases worldwide. Indeed, the major antigenic membrane protein of “*Ca.* P. asteris” (amp) interacts with some cytoskeleton proteins (actin and myosin) and with two subunits of ATP synthase of insect vector species only [12,13].

Identification of insect vector genes differentially expressed upon phytoplasma infection will help to better describe the molecular mechanisms regulating host-pathogen interaction. Gene expression studies based on RT-qPCR quantification of mRNA levels are extensively used in different research fields, but reliability of such an analysis depends on the use of one or more stably expressed housekeeping genes, as internal reference controls [14-16]. Expression variation of insect housekeeping genes has been studied in *Tribolium* beetles infected with fungus [17], in *Rhodnius prolixus* infected with protozoan [18,19], in bees challenged with *Escherichia coli*[20], in planthoppers [21] and whiteflies [22] infected by viruses, but no information is available for phytoplasma vector species or any other leafhopper.

Chrysanthemum yellows phytoplasma (CYP), 16SrI-B (“*Ca*. P. asteris”), is associated with a disease of ornamental plants in north-western Italy, where *Macrosteles quadripunctulatus* Kirschbaum and *Euscelidius variegatus* Kirschbaum (Hemiptera: Cicadellidae) are the most important and efficient vectors [23]. CYP acquisition and transmission efficiencies by both leafhoppers are very high and have been described [24-26]. From previous studies, we know that CYP multiplication is fast and within a few days post acquisition (dpa) the phytoplasma titre can be measured by qPCR in total DNA of infected insects [26] as well as of infected plants [27].

In this work we analysed the stability of a set of putative housekeeping genes in nymphs and adults of two leafhopper vector species infected by CY phytoplasma. The identified reference genes will be useful tools to investigate differential gene expression of leafhopper vectors upon phytoplasma infection and will allow the description of molecular mechanisms regulating insect-phytoplasma relationships and possibly involved in vector specificity.

## Results and discussion

### Isolation of candidate reference genes and sequence analysis

Three housekeeping genes (*ribosomal 18S*, *actin* and *glyceraldehyde-3-phosphate dehydrogenase*, *GAPDH*) as well as genes encoding tropomyosin, an actin-binding protein involved in cytoskeleton organization, and ATP synthase β, a protein involved in specific phytoplasma-host interaction [13], were selected for expression analysis. Due to the absence of sequence information regarding the genomes of the two vector species, coding sequences were obtained from sequencing of cloned *E. variegatus* and *M. quadripunctulatus* amplicons obtained by RT-PCR driven by degenerate primers (Additional file 1: Table S1).

Among the two vector species, *ribosomal 18S*, *actin*, *ATP synthase β*, *GAPDH*, and *tropomyosin* DNA sequence identity values were 99, 97, 89, 87 and 93%, respectively. Corresponding amino acid sequences deduced from *actin*, *ATP synthase β*, *GAPDH*, and *tropomyosin* genes were 100, 99, 98 and 99% similar between the two species, respectively.

*Ribosomal 18*S of *E. variegatus* (EVU15148) and *M. quadripunctulatus* (JX273234) matched in BLASTN with homologous genes of *Flexamia areolata*, *Bothrogonia* sp., *Graphocephala atropunctata*, *Putoniessa rivularis*, and *Tettigella viridis* as first five best matching hits. Sequence identities ranged between 98 and 99%.

*Actin* DNA sequences of *E. variegatus* (HQ451984) and *M. quadripunctulatus* (JX273235) matched in BLASTN with homologous proteins of *Oncometopia nigricans*, *Homalodisca coagulata*, *Mamestra brassicae*, *Manduca sexta*, *Helicoverpa armigera*, *Agrotis ipsilon*, *Papilio polytes*, with identities comprised 92 and 95%. Corresponding amino acid sequences deduced from *actin* gene of these species were at least 99% similar to those of CYP vector species.

*ATP synthase β* DNA sequences of *E. variegatus* (HQ451985) and *M. quadripunctulatus* (JX273236) had greatest homology with *Helicoverpa zea*, *Strongylocentrotus purpuratus*, *Oreochromis niloticus*, *Tetraodon nigroviridis*, *Hemicentrotus pulcherrimus* and *Dendroctonus ponderosae*, with identities varying from 85 to 86%. Since most of DNA matching species were not insects, deduced amino acid ATP synthase β sequences of the two CYP vector species were directly analysed in BLASTP and matched with homologous of *Schistocerca gregaria*, *Pediculus humanus corporis*, *Harpegnathos saltator*, *Dendroctonus ponderosae*, *Camponotus floridanus*, *Apis mellifera* and *Nasonia vitripennis*, with similarities ranging from 97 to 98%.

*GAPDH* gene sequences of *E. variegatus* (JX273239) and *M. quadripunctulatus* (JX273237) matched in BLASTN with homologous genes of *Oncometopia nigricans*, *Laodelphax striatellus*, *Nasonia vitripennis*, *Culex quinquefasciatus*, *Spodoptera litura*, with identities between 79 to 84%. Corresponding amino acid sequences deduced from *GAPDH* genes of these species were 87 to 98% similar to those of CYP vector species.

*Tropomyosin* DNA sequences of *E. variegatus* (JX273240) and *M. quadripunctulatus* (JX273238) blasted with homologous genes from *Periplaneta fuliginosa*, *Periplaneta americana*, *Nasonia vitripennis*, *Blattella germanica*, *Lethocerus indicus*, *Culex quinquefasciatus* and *Bombyx mori*, with identities varying from 79 to 84%. Corresponding amino acid sequences deduced from *tropomyosin* genes of these species were 93 to 98% similar to those of CYP vector species.

For most of the analyzed genes these are the first sequence information for leafhoppers, vectors of economically important plant pathogens.

### Optimization of qPCR assays

To identify the best reference genes, SYBR green qPCR assays were optimized for the transcription profiling of the five genes in the two phytoplasma vector species (Table 1). Two primer pairs, one for each species, were used to amplify *tropomyosin*: TMFw237/Rv461, designed on *E. variegatus* sequence, did not produce any amplification signal from *M. quadripunctulatus* cDNA, due to two mismatches on each oligonucleotide sequence. *M. quadripunctulatus tropomyosin* was therefore amplified by TMFw33/Rv175. Efficiencies of qPCR reactions ranged between 83% and 118% for ActFw832/Rv1021 and TMFw33/Rv175 primer pairs, respectively, whereas correlation coefficients varied from 0.892 to 0.998 for TMFw237/Rv461 and GAPFw632/Rv682, respectively (Table 1). The presence of a single electrophoretic band of the expected size for each target gene (data not shown) and of a single peak in melting curve analysis (Table 1) confirmed the specificity of each primer pair. Differences of less than 1°C were observed among melting peaks of amplicons obtained from most genes of the two insect species, confirming the high similarity of gene sequences. Identical melting profiles were shown by *actin* amplicons. No amplification signal was detected from no-reverse transcribed RNA and from no-template controls.

**Table 1:** Details of the primer pairs used for qPCR

**Target gene**	**Primer name**	**5′-3′sequence**	**Amplicon size (bp)**	**Annealing Temp.**	**Melting peak**	**PCR Efficiency**	**R**^**2**^
*Ribosomal 18S*	Mq Fw	AACGGCTACCACATCCAAGG	98	65°C	81.5°C^§^ 82.2°C^†^	99%	0.982
	Mq Rv2	GCCTCGGATGAGTCCCG					
*Actin*	ActFw832	AAGGACCTGTACGCCAACAC	190	65°C	83°C^§†^	83%	0.987
	ActRv1021	GCTGGAAGGTGGACAGAGAG					
*ATP synthase β*	ATPβFw622	CGCTTTACTCAGGCTGGTTC	171	60°C	84.5°C^§^ 83.5°C^†^	100%	0.995
	ATPβRv792	GTCATCAGCTGGCACGTAGA					
*GAPDH*	GAPFw632	ATCCGTCGTCGACCTTACTG	51	60°C	77.8°C^§^ 77.5°C^†^	99%	0.998
	GAPRv682	TCATCGTAGCTGGCTTCCTTG					
*Tropo-myosin*	TMFw237	AAACGCCGAGAGTGAGGTG	225	60°C	87.1°C^§^	85%	0.892
	TMRv461	AAGAACCGAGCCTCCTTCAG					
	TMFw33	GAAGCTGGAGAAGGACAACG	143	60°C	85.2°C^†^	118%	0.947
	TMRv175	TGTCCAGCTCGTTCTCAATG					

^§^Calculated by amplifying *Euscelidius variegatus* cDNA.

^†^Calculated by amplifying *Macrosteles quadripunctulatus* cDNA.

### Amplification profiles of candidate reference genes

**Figure 1:**
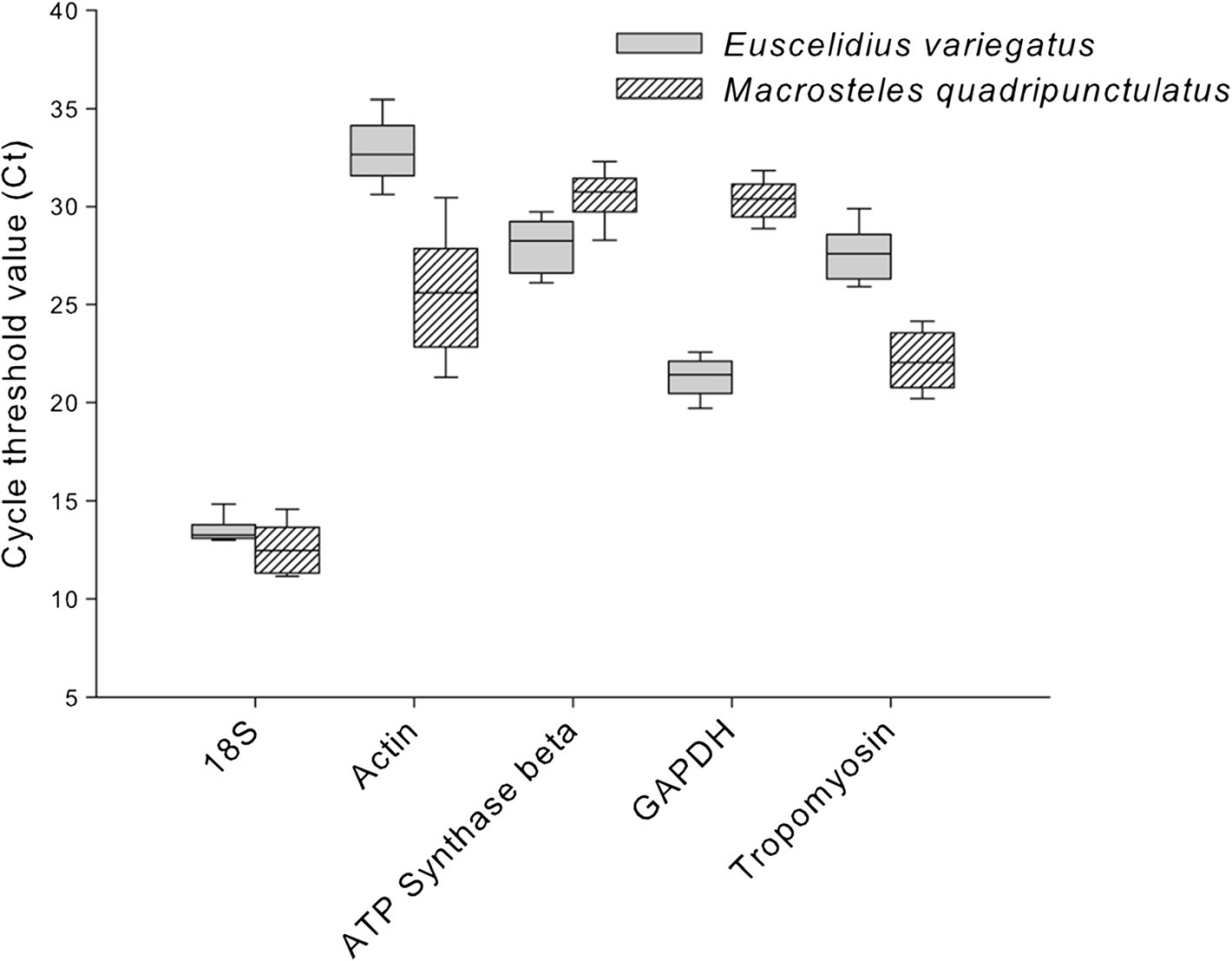
**Amplification profiles of candidate reference genes.** Box plot of qPCR cycle threshold values (Cq) for candidate reference genes in the two phytoplasma vector species. The median is depicted as the line across the box; the box indicates the 25^th^ and 75^th^ percentiles; whiskers represent the 90^th^ and 10^th^ percentiles.

Cycle threshold (Cq) values obtained amplifying the five putative housekeeping genes from the two vector species were plotted (Figure 1). Cq values for the five genes ranged from 12.26 to 38.52 in *E. variegatus* and from 10.25 to 34.03 in *M. quadripunctulatus*. *Ribosomal 18S* showed the lowest Cq values in both species (13.52 ± 0.74, mean Cq ± std. dev. for *E. variegatus*, and 12.65 ± 1.82 for *M. quadripunctulatus*), being expressed at a very high level compared to other protein encoding mRNAs. A similar amplification profile is reported for the *18S* gene of the vector *Delphacodes kuscheli* challenged by *Mal de Rio Cuarto* fijivirus [28]. Amplification of *actin*, *ATP synthase β*, *GAPDH* and *tropomyosin* of *E. variegatus* showed mean Cqs of 32.89 ± 1.84, 28.05 ± 1.45, 21.19 ± 1.04, 27.62 ± 1.42, respectively. Mean Cqs of *M. quadripunctulatus actin*, *ATP synthase β*, *GAPDH* and *tropomyosin* were 25.66 ± 3.25, 30.57 ± 1.45, 30.30 ± 1.04, 22.23 ± 1.77, respectively.

The Cq values obtained for each gene were compared separately for each species in healthy and infective leafhoppers, irrespective of the different sampling time (3, 20 and 40 dpa). A significant difference (Student’s t test, p = 0.032) was found for *M. quadripunctulatus actin* only, which showed higher Cqs in infected leafhoppers (27.17 ± 3.62) than in the healthy ones (24.59 ± 2.55). Consistent with this result, standard deviations of *actin* Cqs were the highest, and higher in *M. quadripunctulatus* (3.25) compared to *E. variegatus* (1.84). In a previous work aimed to identify housekeeping genes in different tissues of the triatomine Chagas’ disease vector *Rhodnius prolixus*, candidate reference genes showing different Cqs were excluded from geNorm and Normfinder analysis [29]. In that case authors argued that the exclusion of genes with significant variation would improve the final results of gene expression stability analysis, as genes with major variation in expression could be selected as good references by geNorm and Normfinder algorithms. In our case instead, *M. quadripunctulatus actin* was indeed identified as the least stable gene by all the softwares and under all the experimental conditions analyzed (Tables 2, 3, 4). Moreover, in a study to select housekeeping genes for expression analyses in different tissues and development stages of honeybee, despite significant differences among Cqs of four candidate genes, geNorm, Normfinder and BestKeeper analyses successfully identified reliable reference genes [30].

**Table 2:** **Reference genes ranked from the most to the least stable considering CYP-infected *Macrosteles quadripunctulatus *samples**

**Ranking order**	**BestKeeper (N = 9)**		**geNorm (N = 12)**		**Normfinder (N = 12)**	
	**Gene name**	**Std dev [± CP]**	**Gene name**	**M value**	**Gene name**	**Stability value**
1	*GAPDH*	0.77	ATP Syn β^*^	**1.868**	*Tropomyosin*^*¶*^	0.708
2	*Tropomyosin*	1.22	***Tropomyosin***	**1.894**	*ATP Syn β*	1.071
3	*18S*	1.24	GAPDH^*^	**1.919**	*GAPDH*^*¶*^	1.151
4	*ATP Syn β*	1.43	***18S***	**2.516**	*18S*	1.566
5	*Actin*	2.13	***Actin***	**2.873**	*Actin*	2.381

^*^Best combination of two genes according with geNorm (average expression stability M = 1.087).

^¶^Best combination of two genes according with Normfinder (stability value = 0.895).

In bold geNorm M values > 1.5.

**Table 3:** **Reference genes ranked from the most to the least stable considering healthy *Macrosteles quadripunctulatus *samples**

**Ranking order**	**BestKeeper (N = 15)**		**geNorm (N = 17)**		**Normfinder (N = 17)**	
		**Gene name**	**Std dev [± CP]**	**Gene name**	**M value**	**Gene name**	**Stability value**
1	*GAPDH*	0.82	ATP Syn β^*^	1.474	*ATP Syn β*^*¶*^	0.450
2	*ATP Syn β*	0.91	GAPDH^*^	**1.773**	*Tropomyosin*^*¶*^	0.897
3	*18S*	1.04	***18S***	**1.968**	*18S*	1.016
4	*Tropomyosin*	1.06	***Tropomyosin***	**2.104**	*GAPDH*	1.085
5	*Actin*	1.80	***Actin***	**2.248**	*Actin*	1.552

^*^Best combination of two genes according with geNorm (average expression stability M = 0.955).

^¶^Best combination of two genes according with Normfinder (stability value = 0.587).

In bold geNorm M values > 1.5.

**Table 4:** **Reference genes ranked from the most to the least stable considering all *Macrosteles quadripunctulatus *samples**

**Ranking order**	**BestKeeper (N = 24)**		**geNorm (N = 29)**		**Normfinder (N = 29)**	
	**Gene name**	**Std dev [± CP]**	**Gene name**	**M value**	**Gene name**	**Stability value**
1	*GAPDH*	0.79	ATP Syn β^*^	**1.706**	*ATP Syn β*	0.529
2	*ATP Syn β*	1.12	GAPDH^*^	**1.923**	*Tropomyosin*^*¶*^	0.551
3	*18S*	1.12	***Tropomyosin***	**2.056**	*18S*^*¶*^	0.691
4	*Tropomyosin*	1.18	***18S***	**2.237**	*GAPDH*	0.824
5	*Actin*	2.15	***Actin***	**2.672**	*Actin*	1.275

^*^Best combination of two genes according with geNorm (average expression stability M = 1.015).

^¶^Best combination of two genes according with Normfinder (stability value = 0.435).

In bold geNorm M values > 1.5.

### Expression stability analysis of candidate reference genes of *Euscelidius variegatus*

To identify the most suitable reference genes to describe the effects of phytoplasma infection in *E. variegatus*, transcript profiles of CYP-infected insects sampled at different times were analysed by BestKeeper [31], geNorm [32] and Normfinder [33] algorithms (Table 5). *GAPDH*, *18S* and *ATP synthase β* were the most stable genes according to BestKeeper, with x-fold change in expression ranging from 1.60 to 1.94 (threshold <2, Additional file 1: Table S2) [31]. Although *actin* and *tropomyosin* showed a Crossing Point (CP) standard deviation above the consistency limit (>1), standard deviations of CP and x-fold for BestKeeper Index of all genes were acceptable (Additional file 1: Table S2). GeNorm M values were lower than 1.5 limit for all the five genes (Table 5). GeNorm and Normfinder ranked *actin* and *tropomyosin* as the most stable genes and *18S* as the most variable (Table 5). Discrepancies among different algorithms were often found when defining housekeeping genes of arthropods subjected to different experimental conditions [20,30,34-37].

**Table 5:** **Reference genes ranked from the most to the least stable considering CYP-infected *Euscelidius variegatus *samples**

**Ranking order**	**BestKeeper (N = 13)**		**geNorm (N = 13)**		**Normfinder (N = 13)**	
	**Gene name**	**Std dev [± CP]**	**Gene name**	**M value**	**Gene name**	**Stability value**
1	*GAPDH*	0.68	Actin^*^	0.963	*Tropomyosin*	0.149
2	*18S*	0.77	Tropomyosin^*^	1.016	*Actin*^*¶*^	0.167
3	*ATP Syn β*	0.96	*ATP Syn β*	1.091	*GAPDH*	0.170
4	*Actin*	1.20	*GAPDH*	1.105	*ATP Syn β*^*¶*^	0.171
5	*Tropomyosin*	1.33	*18S*	1.345	*18S*	0.238

^*^Best combination of two genes according with geNorm (average expression stability M = 0.517).

^¶^Best combination of two genes according with Normfinder (stability value = 0.094).

GeNorm and Normfinder stability analysis considering only healthy *E. variegatus* samples indicated *tropomyosin*, *ATP synthase* β and *GAPDH* as the three most stable genes even if with different ranking order (Table 6). For BestKeeper analysis, one sample was removed due to its under-expression (over 3-fold [31]), *18S* was the most stable gene, with a consistent Cp standard deviation (Table 6), and the five gene index was acceptable (Additional file 1: Table S3).

**Table 6:** **Reference genes ranked from the most to the least stable considering healthy *Euscelidius variegatus *samples**

**Ranking order**	**BestKeeper (N = 16)**		**geNorm (N = 17)**		**Normfinder (N = 17)**	
	**Gene name**	**Std dev [± CP]**	**Gene name**	**M value**	**Gene name**	**Stability value**
1	*18S*	0.31	*Tropomyosin*	1.225	*ATP Syn β*^*¶*^	0.127
2	*Tropomyosin*	1.01	GAPDH^*^	1.296	*Tropomyosin*^*¶*^	0.130
3	*GAPDH*	1.06	ATP Syn β^*^	1.366	*GAPDH*	0.160
4	*Actin*	1.14	*18S*	1.482	*Actin*	0.170
5	*ATP Syn β*	1.46	***Actin***	**1.611**	*18S*	0.176

^*^Best combination of two genes according with geNorm (average expression stability M = 0.823).

^¶^Best combination of two genes according with Normfinder (stability value = 0.074).

In bold geNorm M values > 1.5.

Overall, the five candidate reference genes resulted reliable housekeeping genes to analyze *E. variegatus* insects challenged by phytoplasma infection over time. Indeed, when considering altogether infected and healthy samples collected at different times (Table 7), for all the genes M values calculated by geNorm were below 1.5 limit, and BestKeeper Index was acceptable (Additional file 1: Table S4). *18S* was the most stable gene according to BestKeeper analysis, while the best gene combinations were *GAPDH* and *ATP synthase* β according to geNorm and *actin* and *GAPDH* according to Normfinder.

**Table 7:** **Reference genes ranked from the most to the least stable considering all *Euscelidius variegatus *samples**

**Ranking order**	**BestKeeper (N = 29)**		**geNorm (N = 30)**		**Normfinder (N = 30)**	
	**Gene name**	**Std dev [± CP]**	**Gene name**	**M value**	**Gene name**	**Stability value**
1	*18S*	0.56	*Tropomyosin*	1.143	*Actin*^*¶*^	0.041
2	*GAPDH*	0.89	GAPDH^*^	1.220	*GAPDH*^*¶*^	0.044
3	*Tropomyosin*	1.18	ATP Syn β^*^	1.256	*ATP Syn β*	0.045
4	*ATP Syn β*	1.24	*Actin*	1.366	*Tropomyosin*	0.049
5	*Actin*	1.27	*18S*	1.423	*18S*	0.063

^*^Best combination of two genes according with geNorm (average expression stability M = 0.759).

^¶^Best combination of two genes according with Normfinder (stability value = 0.030).

*GAPDH, ATP synthase* β, *tropomyosin* and *actin* were selected by geNorm and Normfinder among the most stably expressed genes under different sampling time and sanitary conditions, although with different ranking. The computed BestKeeper index for the five putative housekeeping genes was acceptable (Additional file 1: Table S2, S3 and S4), implicating that they all can be used for normalization. A comparative evaluation of the five candidate reference genes by pair-wise correlation revealed a very high correlation (0.698 < r < 0.875, Additional file 1: Table S4) for four of the candidate genes, with the exception of *18S* (r = 0.492, Additional file 1: Table S4). From the above observations, *GAPDH, ATP synthase* β, *tropomyosin* and *actin* should be the target reference genes for successive studies of the effects of CYP infection on *E. variegatus* transcription profile.

### Expression stability analysis of candidate reference genes of *Macrosteles quadripunctulatus*

Transcript profiles of CYP-infected *M. quadripunctulatus* sampled at different times analysed by BestKeeper, geNorm and Normfinder allowed to identify *actin* as the most variable gene according to all algorithms (Table 2). According to BestKeeper, three samples were removed from the analysis due to a 3-fold over or under-expression [31] and *GAPDH* was the most stable gene, being the only one with CP standard deviation below the consistency limit (>1). BestKeeper Index calculated including all the five genes was however acceptable, with standard deviations of CP and x-fold of 0.93 and 1.90, respectively (Additional file 1: Table S5). On the other hand, geNorm M values were above 1.5 limit for all the candidate genes (Table 2). According to Normfinder, *tropomyosin*, *ATP synthase* β and *GAPDH* were the three most stable genes, and the best gene combination was *tropomyosin* and *GAPDH*.

Similar results were obtained analyzing healthy *M. quadripunctulatus* (Table 3), and healthy and CYP-infected *M. quadripunctulatus* (Table 4). *Actin* was the least stable gene following analysis with the three algorithms. *GAPDH* and *ATP synthase* β were the most stable genes according to BestKeeper, with acceptable BestKeeper Index calculated including all the five genes (Additional file 1: Tables S6 and S7), although *GAPDH* always showed low correlation values (0.523 < r < 0.636, Additional file 1: Tables S5, S6, S7). GeNorm indicated *ATP synthase* β and *GAPDH* as the first and second most stable genes, despite M values always nearly above 1.5 limit. *ATP synthase* β and *tropomyosin* were designated by Normfinder as the two most stable genes (Tables 3 and 4).

Taken together these results indicated that, according to BestKeeper and NormFinder, *ATP synthase* β, *tropomyosin* and *GAPDH* were the most stable genes, while for geNorm analyses, the five selected genes were not stable enough as references for *M. quadripunctulatus* transcript profiling upon CY phytoplasma infection. In previous studies, this species has been described as the most susceptible to CYP infection in terms of pathogen multiplication rate [26] and reduced longevity of infected insects [38] compared to *E. variegatus* and *Euscelis incisus*, both CYP vectors. Moreover, *M. quadripunctulatus* has a faster development and a shorter life span than the other two vector species, and consistently become infective after a shorter latent period than *E. variegatus* (18 d vs 30 d, respectively) [23,26]. Moreover, the analysis of *M. quadripunctulatus* candidate reference genes with geNorm, considering different sampling dates separately (Table 8), showed that, at the first sampling date (3 dpa), all genes but *18S* had an acceptable M value, at the second date (20 dpa), only two genes (*ATP Synthase* β and *GAPDH*) remained below 1.5 limit, whereas at the last sampling (40 dpa), no genes were acceptable as references. The high phytoplasma concentration and the instability of genes involved in different metabolic pathways at late stages of infection are consistent with the reduced fitness parameters observed for this vector species.

**Table 8:** **Reference genes ranked by geNorm in *Macrosteles quadripunctulatus *individuals sampled at different times after acquisition**

**Ranking order**	**Time 1-3dpa (N = 13)**		**Time 2-20dpa (N = 8)**		**Time 3-40dpa (N = 8)**	
	**Gene name**	**M value**	**Gene name**	**M value**	**Gene name**	**M value**
1	Tropomyosin^*^	0.826	*ATP Syn β*^*¶*^	1.440	***GAPDH***^*§*^	**1.923**
2	*ATP Syn β*	0.903	*GAPDH*^*¶*^	1.498	***ATP Syn β***^*§*^	**1.965**
3	Actin^*^	0.916	***Tropomyosin***	**1.711**	***18S***	**2.598**
4	*GAPDH*	1.133	***18S***	**2.126**	***Actin***	**2.803**
5	***18S***	**1.721**	***Actin***	**2.294**	***Tropomyosin***	**2.889**

^*^Best combination of two genes considering individuals sampled at 3 dpa (average expression stability M = 0.394).

^¶^Best combination of two genes considering individuals sampled at 20 dpa (average expression stability M = 0.806).

^§^Best combination of two genes considering individuals sampled at 40 dpa (average expression stability M = 0.864).

In bold geNorm M values > 1.5.

## Conclusions

In this study a validation of candidate reference genes was performed to identify housekeepings for transcript profiling of two leafhopper vector species infected by CY phytoplasma.

According to three analytic methods, all five genes of *E. variegatus* were stable enough to serve as references, and *ATP synthase* β, *GAPDH*, *actin* and *tropomyosin* were the most stable ones. Leafhopper ATP synthase β and actin proteins interact with the major antigenic phytoplasma membrane protein and probably are involved in determining vector specificity [12,13]. Despite these interactions, there are no evidences of ATP synthase β and actin deregulation in insect proteome, and indeed their transcript profiles were stable upon phytoplasma infection. On the other hand, the situation with *M. quadripunctulatus* was less clear, and *actin* was always the least stable gene upon time and infection. According to BestKeeper and Normfinder, *ATP synthase* β, *tropomyosin* and *GAPDH* were the most stable genes upon time and infection, but, according to geNorm, none of the candidate genes was acceptable as reference upon the entire experimental period. GeNorm analysis at each sampling date identified stable reference genes only for early stages of infection.

Reference genes to study effect of phytoplasma infection on gene expression are species specific and need to be evaluated under different experimental conditions.

The identified reference genes, besides adding new insights into the transcriptome of the poorly characterized phytoplasma vectors, are useful tools to further investigate differential gene expression of leafhoppers upon phytoplasma infection.

## Methods

### Phytoplasma strain, insect vectors and experimental samples

Chrysanthemum yellows phytoplasma (CYP, 16Sr-IB), belonging to “*Candidatus* Phytoplasma asteris” [39] was originally isolated from *Argyranthemum frutescens* (L.) Schultz-Bip in Liguria, Italy, and it was maintained by insect transmission on daisy, *Chrysanthemum carinatum* Schousboe. Healthy colonies of *Euscelidius variegatus* and *Macrosteles quadripunctulatus*, vectors of CYP [23], were maintained on oat, *Avena sativa* L. in growth chambers at 25°C and with a photoperiod of 16:8 (L:D) h.

Healthy and CYP-infected insects analysed for gene expression were from the same colony and belonged to the same unique generation. To this purpose, approximately 200 fecundated adult females of each species were allowed to lay eggs on oat plants for 24 h. About 30 fourth instar nymphs were caged on CYP-infected daisies for an acquisition access period (AAP) of 7 days and then transferred to healthy oat plants for the latent period; the remaining insects, not exposed to infected plants, were used as healthy samples. Total RNAs were extracted from healthy and CYP-infected insects collected at 3, 20 and 40 days after the start of the AAP. Five to ten insects for each category (healthy and CYP-infected) of the two species were taken at each sampling date. In the end, 30 (17 healthy and 13 CYP-infected) *E. variegatus* and 29 (17 healthy and 12 CYP-infected) *M. quadripunctulatus* were subjected to stability analysis to determine putative reference genes.

### RNA extraction, quality control and reverse transcription

For gene isolation purpose, total RNAs were extracted from batches of five *E. variegatus* and ten *M. quadripunctulatus* healthy adults with TRIzol reagent (Life Technologies) and reverse transcribed to cDNA with MuLV Reverse Transcriptase and Random Hexamers (Applied Biosystems) following manufacturer’s recommendations and 1 μl was used in PCR with degenerated primers.

For gene expression analysis, total RNA was extracted from healthy or CYP-infected single insects with TRIzol reagent (Life Technologies) and treated with DNase I (Applied Biosystems) following manufacturer’s recommendations. The RNA concentration was measured with a NanoDrop 1000 spectrophotometer (Thermo Fisher Scientific), the RNA purity was checked by the 260/280 nm ratio (accepted values ranged between 1.8 and 2), and the RNA integrity was determined by denaturing agarose gel (1.2%) electrophoresis after ethidium bromide staining. Single insect RNA samples were then diluted in DEPC H_2_O to a final concentration of 10 ng/μl and 10 μl were reverse transcribed to cDNA with High Capacity cDNA Reverse Transcription Kit with Random Hexamers (Applied Biosystems) following manufacturer’s recommendations. Resulting cDNAs were diluted and analysed in qPCR to evaluate expression stability of candidate reference genes.

### Gene isolation and sequence analysis

For gene isolation purpose, *E. variegatus* and *M. quadripunctulatus* cDNAs were amplified with degenerated primers designed on the consensus sequences of the five putative reference genes obtained by aligning coding sequences of several insect species available in genbank (Additional file 1: Table S1). PCR products, corresponding to partial sequences of the five putative housekeeping genes, were gel-purified by Geneclean (MP Biomedicals), cloned into pGemT Easy vector (Promega), and sequenced by BMR Genomics (Padova, Italia). Sequences were assembled, *in silico* translated, and analysed by Mega5 software [40].

### qPCR assays

The minimum information for publication of qPCR experiments (MIQE) guidelines were followed [41]. To determine amplification efficiencies and specificity of each primer pairs used in qPCR assays, 10-fold cDNA dilution series of each insect species were used, from undiluted to 1:1000. To exclude DNA contamination, non reverse transcribed RNA samples were also included in the analysis.

For expression analysis of the five candidate reference genes, all the experimental samples for each species were processed in the same reverse transcription event, the obtained cDNAs were diluted 1:2 in ddH_2_O and 2 μl were used in qPCR within three days. Each sample was analyzed in duplicate in 96 well plate in a total reaction of 20 μL, containing 300 nM of each primer, 1X SYBR Green PCR Master Mix (Applied Biosystems) and 2 μL of diluted cDNA. All the experimental samples of each insect species were amplified in five different qPCR plates, one for each primer pair, in order to amplify the same gene from all the samples in the same reaction. No template controls and a pooled cDNA, including equimolar amount of each samples, were always included in each plate. Reactions were performed on a StepOnePlus Real Time PCR System thermal cycler (Applied Biosystems) using the following cycling conditions: 95°C for 3 min and 40 cycles at 95°C for 15 s and 60°C or 65°C (*18S* and *actin*) for 1 min. The specificity of the PCR products was verified by melting curve analysis for all samples.

### Data analysis

Cq values were submitted to Grubb’s test for outlier detection [42], and compared between different conditions using one-way ANOVA or Student’s t-test, depending on the number of experimental groups analysed. These statistical analyses were performed using SigmaPlot 11 (Systat Software).

To calculate the stability of candidate reference genes the algorithms BestKeeper [31], geNorm [32], and NormFinder [33] were used. BestKeeper uses Cq values to determine the fold variation of different target and housekeeping genes, combining the latter ones into an index. Reference genes can be ordered from the most stable, exhibiting the lowest variation expressed as standard deviation (SD) and coefficient of variance (CV), to the least stable, exhibiting the highest variation.

Raw Cq values were transformed into relative quantities according with geNorm and Normfinder requirements. GeNorm calculates for each reference gene a stability measure (M value) based on the geometric mean of all studied genes and the pairwise variation. NormFinder calculates a stability value and indicates the best combination of two genes in terms of expression stability among a set of candidate reference genes. The lower are the geNorm M value and the NormFinder stability index, the more stable is the gene.

## Abbreviations

GAPDH: Glyceraldehyde-3-phosphate dehydrogenase; CYP: Chrysanthemum yellows phytoplasma; dpa: Days post acquisition; Cq: Cycle threshold; CP: Crossing Point; AAP: Acquisition access period

## Competing interests

The authors declare that they have no competing interests.

## Authors’ contributions

LG performed the experimental procedures and data analysis. DB reared insects. CM supervised the research. LG wrote the manuscript, CM and DB assisted in revising it and provided helpful discussions. All authors read and approved the final manuscript.

## Supplementary Material

Additional file 1: Table S1. Details of primers used to amplify and sequence insect genes. **Table S2:** Descriptive statistics calculated by BestKeeper considering CYP-infected *Euscelidius variegatus* samples. Table S3: Descriptive statistics calculated by BestKeeper considering healthy *Euscelidius variegatus* samples. **Table S4:** Descriptive statistics calculated by BestKeeper considering all *Euscelidius variegatus* samples. **Table S5:** Descriptive statistics calculated by BestKeeper considering CYP-infected *Macrosteles quadripunctulatus* samples. **Table S6:** Descriptive statistics calculated by BestKeeper considering healthy *Macrosteles quadripunctulatus* samples. **Table S7:** Descriptive statistics calculated by BestKeeper considering all *Macrosteles quadripunctulatus* samples. Click here for file
