# Supplementary material for: Selection of reference genes from two leafhopper species challenged by phytoplasma infection, for gene expression studies by RT-qPCR
Source: BMC Res Notes. 2013 Oct 11;6:409. doi: 10.1186/1756-0500-6-409 (PMC3852609; doi:10.1186/1756-0500-6-409)
Supplement: Additional file 1: Table S1. — Details of primers used to amplify and sequence insect genes. Table S2: Descriptive statistics calculated by BestKeeper considering CYP-infected Euscelidius variegatus samples. Table S3: Descriptive statistics calculated by BestKeeper considering healthy Euscelidius variegatus samples. Table S4: Descriptive statistics calculated by BestKeeper considering all Euscelidius variegatus samples. Table S5: Descriptive statistics calculated by BestKeeper considering CYP-infected Macrosteles quadripunctulatus samples. Table S6: Descriptive statistics calculated by BestKeeper considering healthy Macrosteles quadripunctulatus samples. Table S7: Descriptive statistics calculated by BestKeeper considering all Macrosteles quadripunctulatus samples. [file 1756-0500-6-409-S1.docx]

**Table S1: Details of primers used to amplify and sequence insect genes**

| **Target gene** | **Primer name** | **5´-3´sequence** | **Position from ATG** | | **Organisms aligned for primer design** | **Accession number** |
| --- | --- | --- | --- | --- | --- | --- |
| *18S* | 18SFw1 | GTTGCGGTTAAAAAGCTCGT | 37 | | *Euscelidius variegatus* | EVU15148 |
|  | 18SRv1 | GTACAAAGGGCAGGGACGTA | 1158 | |  |  |
| *Actin* | ActFw1 | ATGTGTGACGA**W**GA**KR**T**W**GC**M**GC | 1 | | *Culex pipiens pipiens*  *Acyrthosiphon pisum*  *Toxoptera citricida* | DQ385449  NM_001142636  AY737550 |
|  | ActFw750 | GACGGACAGGTCATCACCATCGG | 732 | |  |  |
|  | ActRv376 | GAAGCGTACAGGGAGAGCAC | 376 | |  |  |
|  | ActRv750 | CCGATGGTGATGACCTGTCCGTC | 756 | |  |  |
|  | ActRv1110 | CC**D**GGGCCGGA**Y**TCGTCGT | 1106 | |  |  |
| *ATP synthase β* | ATPβFw220 | CAGCA**Y**TTGGG**W**GAAAA**Y**AC | 284 | | *Nasonia vitripennis*  *Acyrthosiphon pisum*  *Drosophila melanogaster*  *Apis mellifera* | NM_001159894  NM_001126173  NM_166808  XM_624153 |
|  | ATPβRv1100 | GGATC**B**ACAGC**W**GG**R**TA**R**ATACC | 1200 | |  |  |
| *GAPDH* | GAPFw3 | ATG**WSR**AA**S**AT**Y**GG**W**AT**Y**AACGG | 1 | | *Nasonia vitripennis*  *Laodelphax striatellus*  *Culex quinquefasciatus*  *Oncometopia nigricans*  *Spodoptera litura* | XM_001607424  HQ385974  XM_001846997  AY725786  HQ012003 |
|  | GAPRv3 | TTA**R**TC**Y**TTG**SWY**TG**S**ATGTA**Y**TTGAT | 999 | |  |  |
| *Tropo-myosin* | TmFw1 | ATGGACGC**S**ATCAAGAAGAAAATGC | | 1 | *Myzus persicae*  *_Acyrtosiphon pisum*  *Toxoptera citricida*  *Culex quinquefasciatus*  *Aedes aegypti*  *Apis mellifera*  *Bombyx mori* | AY714063 NM_001246093  AY737537  XM_001864204  XM_001655903  XM_392125  DQ443311 |
|  | TmFw49 | ATGAAGCTGGAGAAGGACA | | 31 |  |  |
|  | TmRv503 | TTACGCGCTACCTCATCATATT | | 503 |  |  |
|  | TmRv752 | TCGTCTTCGAG**Y**CTGTCGACTTCC | | 776 |  |  |
|  | TmRv876 | TGTA**Y**TTCTC**Y**TTCTC | | 802 |  |  |

**Table S2: Descriptive statistics calculated by BestKeeper considering CYP-infected *Euscelidius variegatus* samples**

|  | ***18S*** | ***Actin*** | ***ATP Syn β*** | ***GAPDH*** | ***Tropomyosin*** | **BestKeeper** |
| --- | --- | --- | --- | --- | --- | --- |
| **n** | 13 | 13 | 13 | 13 | 13 | 13 |
| **geo Mean [CP]** | 13.79 | 33.25 | 28.01 | 21.17 | 27.84 | 23.76 |
| **ar Mean [CP]** | 13.82 | 33.28 | 28.03 | 21.18 | 27.88 | 23.77 |
| **min [CP]** | 12.26 | 31.43 | 25.32 | 19.91 | 25.59 | 22.69 |
| **max [CP]** | 15.42 | 35.58 | 29.27 | 22.59 | 30.12 | 25.15 |
| **std dev [± CP]** | 0.77 | 1.20 | 0.96 | 0.68 | 1.33 | 0.75 |
| **CV [% CP]** | 5.58 | 3.62 | 3.43 | 3.22 | 4.76 | 3.16 |
| **min [x-fold]** | -2.86 | -3.01 | -6.44 | -2.38 | -4.01 | 2.05 |
| **max [x-fold]** | 3.07 | 4.08 | 2.40 | 2.66 | 4.06 | 2.57 |
| **std dev [± x-fold]** | 1.70 | 2.29 | 1.94 | 1.60 | 2.49 | 1.66 |
| **coeff. of corr. [r]** | 0,550 | 0,937 | 0,731 | 0,582 | 0,934 |  |
| **p-value** | 0,052 | 0,001 | 0,005 | 0,037 | 0,001 |  |

**Table S3: Descriptive statistics calculated by BestKeeper considering healthy *Euscelidius variegatus* samples**

|  | ***18S*** | | ***Actin*** | ***ATP Syn β*** | ***GAPDH*** | ***Tropomyosin*** | **BestKeeper** |
| --- | --- | --- | --- | --- | --- | --- | --- |
| **n** | | 16 | 16 | 16 | 16 | 16 | 16 |
| **geo Mean [CP]** | | 13.30 | 32.20 | 27.95 | 21.14 | 27.25 | 23.32 |
| **ar Mean [CP]** | | 13.31 | 32.23 | 28.00 | 21.17 | 27.28 | 23.34 |
| **min [CP]** | | 12.53 | 30.40 | 25.50 | 19.25 | 25.77 | 21.97 |
| **max [CP]** | | 14.60 | 35.48 | 30.80 | 23.21 | 30.15 | 24.91 |
| **std dev [± CP]** | | 0.31 | 1.14 | 1.46 | 1.06 | 1.01 | 0.77 |
| **CV [% CP]** | | 2.29 | 3.54 | 5.22 | 4.99 | 3.71 | 3.31 |
| **min [x-fold]** | | -1.70 | -2.97 | -5.48 | -3.68 | -2.49 | 2.49 |
| **max [x-fold]** | | 2.45 | 7.24 | 7.23 | 4.15 | 5.93 | 2.93 |
| **std dev [± x-fold]** | | 1.23 | 2.19 | 2.73 | 2.07 | 2.01 | 1.69 |
| **coeff. of corr. [r]** | | 0,354 | 0,816 | 0,921 | 0,812 | 0,810 |  |
| **p-value** | | 0,177 | 0,001 | 0,001 | 0,001 | 0,001 |  |

**Table S4: Descriptive statistics calculated by BestKeeper considering all *Euscelidius variegatus* samples**

|  | ***18S*** | ***Actin*** | ***ATP Syn β*** | ***GAPDH*** | ***Tropomyosin*** | **BestKeeper** |
| --- | --- | --- | --- | --- | --- | --- |
| **n** | 29 | 29 | 29 | 29 | 29 | 29 |
| **geo Mean [CP]** | 13.52 | 32.67 | 27.98 | 21.15 | 27.51 | 23.51 |
| **ar Mean [CP]** | 13.54 | 32.70 | 28.01 | 21.18 | 27.55 | 23.53 |
| **min [CP]** | 12.26 | 30.40 | 25.32 | 19.25 | 25.59 | 21.97 |
| **max [CP]** | 15.42 | 35.58 | 30.80 | 23.21 | 30.15 | 25.15 |
| **std dev [± CP]** | 0.56 | 1.27 | 1.24 | 0.89 | 1.18 | 0.78 |
| **CV [% CP]** | 4.14 | 3.89 | 4.42 | 4.20 | 4.28 | 3.30 |
| **min [x-fold]** | -2.38 | -3.94 | -6.30 | -3.71 | -3.28 | 2.84 |
| **max [x-fold]** | 3.71 | 5.81 | 7.10 | 4.11 | 5.04 | 3.03 |
| **std dev [± x-fold]** | 1.47 | 2.40 | 2.34 | 1.84 | 2.25 | 1.69 |
| **coeff. of corr. [r]** | 0,492 | 0,875 | 0,818 | 0,698 | 0,872 |  |
| **p-value** | 0,007 | 0,001 | 0,001 | 0,001 | 0,001 |  |

**Table S5: Descriptive statistics calculated by BestKeeper considering CYP-infected *Macrosteles quadripunctulatus* samples**

|  | ***18S*** | ***Actin*** | ***ATP Syn β*** | ***GAPDH*** | ***Tropomyosin*** | **BestKeeper** |
| --- | --- | --- | --- | --- | --- | --- |
| **n** | 9 | 9 | 9 | 9 | 9 | 9 |
| **geo Mean [CP]** | 12.43 | 25.60 | 30.27 | 29.96 | 22.08 | 22.95 |
| **ar Mean [CP]** | 12.51 | 25.75 | 30.32 | 29.98 | 22.14 | 22.99 |
| **min [CP]** | 10.43 | 20.99 | 27.67 | 27.93 | 19.70 | 20.41 |
| **max [CP]** | 15.28 | 29.23 | 33.08 | 31.41 | 25.00 | 24.62 |
| **std dev [± CP]** | 1.24 | 2.13 | 1.43 | 0.77 | 1.22 | 0.93 |
| **CV [% CP]** | 9.91 | 8.26 | 4.72 | 2.55 | 5.53 | 4.04 |
| **min [x-fold]** | -3.97 | -16.23 | -6.05 | -4.05 | -6.41 | 5.81 |
| **max [x-fold]** | 7.13 | 8.98 | 7.02 | 2.72 | 9.71 | 3.18 |
| **std dev [± x-fold]** | 2.35 | 4.32 | 2.68 | 1.69 | 2.32 | 1.90 |
| **coeff. of corr. [r]** | 0,523 | 0,783 | 0,780 | 0,514 | 0,866 |  |
| **p-value** | 0,149 | 0,013 | 0,013 | 0,156 | 0,003 |  |

**Table S6: Descriptive statistics calculated by BestKeeper considering healthy *Macrosteles quadripunctulatus* samples**

|  | ***18S*** | ***Actin*** | ***ATP Syn β*** | ***GAPDH*** | ***Tropomyosin*** | **BestKeeper** |
| --- | --- | --- | --- | --- | --- | --- |
| **n** | 15 | 15 | 15 | 15 | 15 | 15 |
| **geo Mean [CP]** | 12.30 | 24.01 | 30.51 | 30.45 | 21.66 | 22.64 |
| **ar Mean [CP]** | 12.36 | 24.10 | 30.53 | 30.47 | 21.70 | 22.66 |
| **min [CP]** | 10.25 | 21.17 | 28.27 | 28.70 | 20.20 | 20.87 |
| **max [CP]** | 14.57 | 27.44 | 32.28 | 31.90 | 24.05 | 24.06 |
| **std dev [± CP]** | 1.04 | 1.80 | 0.91 | 0.82 | 1.06 | 0.92 |
| **CV [% CP]** | 8.40 | 7.46 | 2.99 | 2.70 | 4.89 | 4.08 |
| **min [x-fold]** | -4.08 | -5.58 | -4.73 | -3.34 | -3.13 | 3.40 |
| **max [x-fold]** | 4.76 | 7.94 | 3.43 | 2.71 | 6.42 | 2.67 |
| **std dev [± x-fold]** | 2.04 | 3.45 | 1.87 | 1.76 | 2.08 | 1.90 |
| **coeff. of corr. [r]** | 0,636 | 0,858 | 0,922 | 0,395 | 0,820 |  |
| **p-value** | 0,011 | 0,001 | 0,001 | 0,145 | 0,001 |  |

**Table S7: Descriptive statistics calculated by BestKeeper considering all *Macrosteles quadripunctulatus* samples**

|  | ***18S*** | ***Actin*** | ***ATP Syn β*** | ***GAPDH*** | ***Tropomyosin*** | **BestKeeper** |
| --- | --- | --- | --- | --- | --- | --- |
| **n** | 24 | 24 | 24 | 24 | 24 | 24 |
| **geo Mean [CP]** | 12.35 | 24.60 | 30.42 | 30.27 | 21.82 | 22.75 |
| **ar Mean [CP]** | 12.41 | 24.72 | 30.45 | 30.28 | 21.86 | 22.78 |
| **min [CP]** | 10.25 | 20.99 | 27.67 | 27.93 | 19.70 | 20.41 |
| **max [CP]** | 15.28 | 29.23 | 33.08 | 31.90 | 25.00 | 24.62 |
| **std dev [± CP]** | 1.12 | 2.15 | 1.12 | 0.79 | 1.18 | 0.96 |
| **CV [% CP]** | 8.99 | 8.69 | 3.69 | 2.61 | 5.42 | 4.20 |
| **min [x-fold]** | -4.22 | -8.86 | -6.71 | -5.01 | -5.23 | 5.06 |
| **max [x-fold]** | 7.55 | 16.44 | 6.33 | 3.08 | 11.90 | 3.65 |
| **std dev [± x-fold]** | 2.16 | 4.39 | 2.17 | 1.72 | 2.26 | 1.94 |
| **coeff. of corr. [r]** | 0,586 | 0,814 | 0,818 | 0,391 | 0,845 |  |
| **p-value** | 0,003 | 0,001 | 0,001 | 0,059 | 0,001 |  |
